# The value of completion residual lung resection in ipsilateral recurrent non-small cell lung cancer

**DOI:** 10.3389/fsurg.2022.990282

**Published:** 2022-11-09

**Authors:** Yong Yang, Yichao Wang, Ziwei Wan, Xiong Qin, Yuming Zhu, Bingyong Sheng, Xiaogang Zhao

**Affiliations:** ^1^ Department of Thoracic Surgery, Shanghai Pulmonary Hospital, Tongji University School of Medicine, Shanghai, China; ^2^ Department of Oncology, Yueyang Hospital of Integrated Traditional Chinese and Western Medicine, Shanghai University of Traditional Chinese Medicine, Shanghai, China; ^3^ Department of Radiology, Shanghai Pulmonary Hospital, Tongji University School of Medicine, Shanghai, China

**Keywords:** recurrence, lung cancer, outcome, completion pulmonary resection, postrecurrence survival, overall survival

## Abstract

**Background:**

Recurrence is one of the most important challenges to manage lung cancer. Selected patients might be candidates for resection. This study assessed the outcomes and hazard factors of patients after completion of lung resection for recurrence, focusing specifically on postrecurrence survival (PRS) and overall survival (OS) after surgery.

**Methods:**

This retrospective study enrolled 63 patients who underwent complete pulmonary resection for recurrence between January 2015 and December 2018. Inclusion criteria include potentially curative first resection for primary lung cancer, histologically proven recurrent or new malignancy, and complete pathological report after both operations. PRS and OS were assessed and the influence of patient and treatment features on these endpoints was evaluated.

**Results:**

Most of the patients recurred at stage IIIA, and nearly three-fourth received complete pneumonectomy. The overall 2- and 5-year survival rates were 95% and 75%, whereas the overall 2- and 5-year postrecurrence survival rates were 55% and 36%, respectively. No patient died within 30 or 90 days after completion of residual lung resection, and no serious complications occurred during follow-up. Upon selection of clinically important variables by the Cox proportional hazards regression model, the r-stage [hazard ratio (HR), 3.35; 95% CI, 1.11–10.10; *P* = 0.03] and stage of primary tumor (HR, 6.26; 95% CI, 2.00–19.55; *P* < 0.01) were hazard factors for PRS and OS respectively.

**Conclusions:**

Complete pulmonary resection is an acceptable option in selected patients with recurrent lung cancer after surgery. The patients with r-stage earlier than IIIA may benefit from completion pulmonary resection but not IIIB. Completion pneumonectomy failed to significantly prolong the OS. The OS in the enrolled cases was mainly affected by the p-TNM stage assessed by the first resection for primary lung cancer.

## Introduction

Non-small cell lung cancer (NSCLC) is a common malignancy with poor prognosis. One of the main causes of poor long-term survival is postoperative tumor recurrence (1). It was reported that locoregional recurrence occurs in 4.6%–24% of patients after complete resection (2). It was found that surgery may be possible in up to 4% of patients with recurrence (3). Meanwhile, following the early detection and treatment of the malignant lesion in these years, several cases received lobectomy or segmentectomy for the first surgery. Consequently, an increasing number of patients experienced ipsilateral recurrence. Subsequent completion resection in these patients may include pneumonectomy, lobectomy or bilobectomy, and even sublobar resection. Other therapeutic options are definitive targeted therapy, chemotherapy, immunotherapy, and radiation therapy, either alone or combined (4, 5).

The selection of the best candidates for this kind of surgical resection is not univocal and still lacks sufficient evidence. There are only a few large-scale studies with more attention on the risk of this operation instead of the oncological benefit (6). The purpose of the present study is to identify the role and applicability of complete resection for ipsilateral recurrent NSCLC and identify clinicopathological factors associated with survival and the risk of recurrence after complete resection.

## Materials and methods

### Study population

With the Ethics Committee of Shanghai Pulmonary Hospital's approval, we retrospectively analyzed a cohort of patients who underwent complete pulmonary resection for an ipsilateral recurrence after a radical operation at the Shanghai Pulmonary Hospital between January 2015 and December 2018.

Patients affected by new lesions after surgery suspected as new primaries or recurrence received positron emission tomography (PET)-computed tomography (CT), brain MRI, and/or needle biopsy depending on the lesion position after diagnostic anti-inflammatory therapy for 2 weeks. Endobronchial ultrasound-guided transbronchial needle aspiration was performed on PET-CT or CT suspicious enlarged or metastatic N2 cases. If the lesion was confirmed as malignancy without other metastasis, the patient was evaluated to receive surgery, radiotherapy, or chemotherapy. Inclusion criteria for completion of pulmonary resection include potentially curative first resection for primary lung cancer, histologically proved recurrence, and/or CT suspicion of malignancy at the ipsilateral hilar or other parenchymal levels (7), no preoperative cN2 disease, and no distant metastases, detailed postsurgical histopathological report, and exact data about the treatment outcome at the time of the last contact with patients or their families. Exclusion criteria for complete pulmonary resection include secondary primary lung cancer or benign tumor diagnosed by pathology, and positive precedent history of malignancy other than lung cancer. The resection extension was chosen based on the cardiopulmonary function, the location of the tumor and its size, and the aim of both radical resection and avoidance of potential postoperative lung expansion deficit. In this series, there were no patients suffering from a middle lobe recurrence with a favorable local situation in terms of oncological radicality, in which it would be possible to avoid completion pneumonectomy.

### Completion residual lung resection

The preoperative lung function and risk assessment before the second resection were performed according to the European Respiratory Society (ERS) and the European Society of Thoracic Surgery (ESTS) criteria (8). Spirometry was performed routinely to evaluate the diffusing capacity of the lung for carbon monoxide (DLCO) and capillary blood gas. The circulatory system was assessed based on electrocardiography and echocardiography; additional examinations were performed in individual cases, such as a 24-h Holter electrocardiogram. Hyposaturation in the arterial blood at rest was a contraindication for surgery.

The patients who underwent video-assisted thoracoscopic surgery (VATS) lung resection or open surgery in our institute were placed in a lateral position on the operating table under general anesthesia with selective lung ventilation. Anterolateral mini-thoracotomy (4–5 cm) was performed in the fourth or fifth intercostal space for VATS surgery. Thoracotomy completion lung resection was performed *via* the previous wound extended to a 7–13 cm incision. VATS was mostly performed in sublobectomy completion resection, whereas an open approach was applied for pneumonectomy cases. In addition, the patients who suffered from severe thoracic adhesion or fibrotic hilar structure also received open surgery.

The policy for lymphadenectomy during lung resection in all lung cancer patients comprised systematic lymphadenectomy from the supreme mediastinal level down to the pulmonary ligament level. The minimal quality requirement was at least three different nodal groups removed, including those removed en blocs with the specimen.

### Follow-up

All data were obtained from our institutional databases. Data retrieved included age (at first surgery and recurrence), sex, tumor location and size, genetic mutation, stage of the index tumor, adjuvant or neoadjuvant treatment, and comorbidity. For staging, the 8th edition of the TNM classification for lung cancer was used (9). Additional surgical information included the date of surgery, type of resection, extent of lymph node dissection, in-hospital complications, and length of stay in the intensive care unit (ICU) and in the hospital. During follow-up after surgery, a CT scan was routinely performed every 3–4 months in the first 2 years, every 6 months in the 3rd to 5th year, and thereafter every year. A CT scan and/or PET-CT scan, with or without other routine examinations, was adopted in patients with clinical suspicion of progression or recurrence of the disease.

### Statistical analysis

Statistical analysis was performed using the R package (version 4.1.3, R Core team, Vienna, 2019). Median follow-up times were estimated using the Kaplan–Meier method. Postrecurrence survival (PRS) and overall survival (OS) distributions were analyzed using the Kaplan–Meier method. PRS was calculated from the date of complete surgery to the date of progression of the disease (recurrence or metastasis). OS was defined as the time between the date of surgery completion and death of any cause. For further analysis, backward stepwise selection using the Akaike information criterion (AIC) in the Cox proportional hazards regression model was used to assess the associations of relevant clinicopathological variables with PRS and OS. Hazard ratios (HRs) were presented with their 95% CIs. All tests were two-sided, and *P* < 0.05 was considered statistically significant.

## Results

### Patients and tumors characteristics

**Figure 1:**
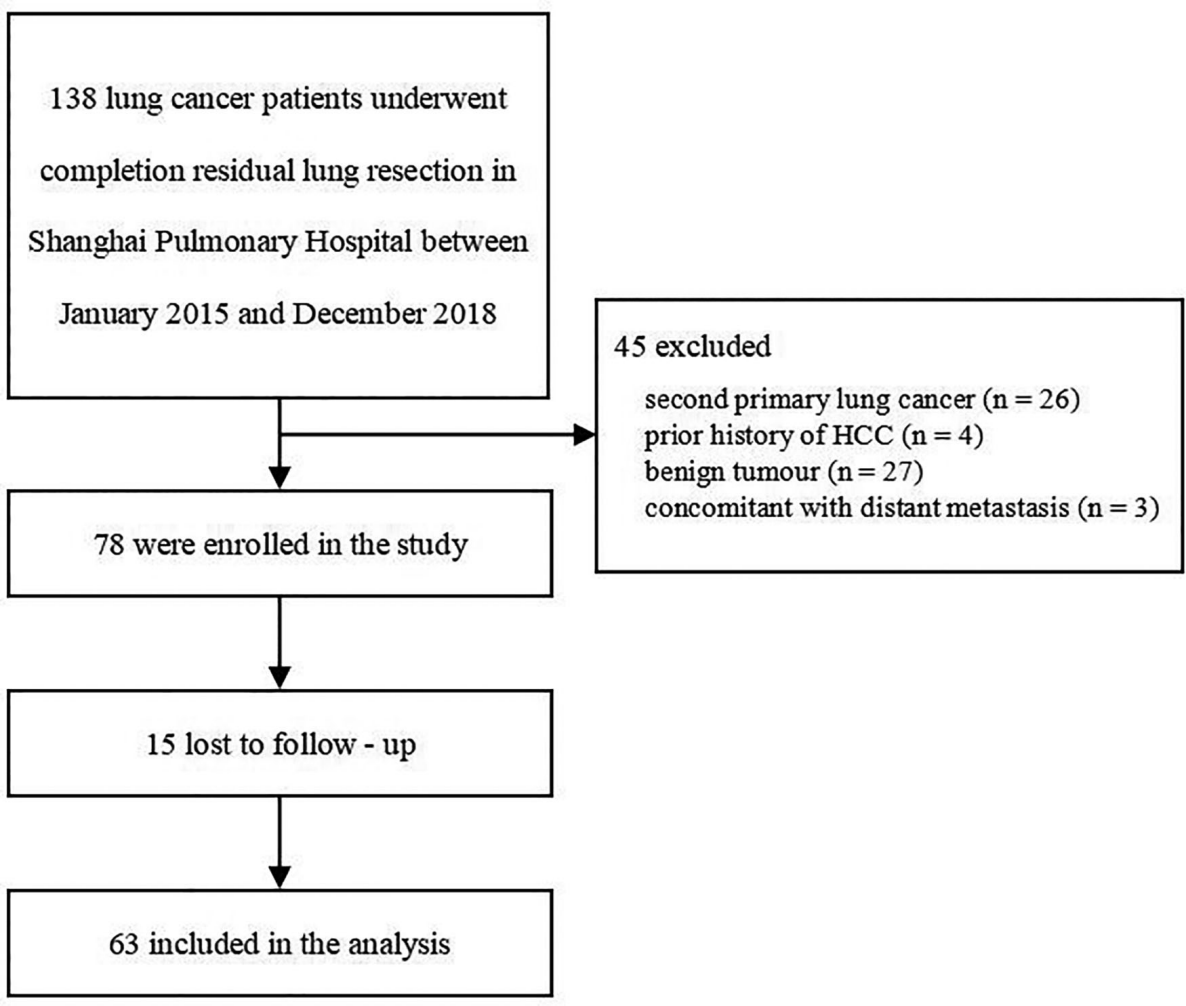
Flowchart demonstrating patient inclusion and exclusion.

A total of 138 lung cancer patients underwent completion residual lung resection at the Shanghai Pulmonary Hospital between January 2015 and December 2018, and 78 patients were enrolled in the study. Sixty patients were excluded because of second primary lung cancer (*n* = 26), history of hepatocellular carcinoma (HCC) (*n* = 4), benign tumor (*n* = 27), or concomitant with distant metastasis (*n* = 3). Of the enrolled patients, 15 patients were lost to follow-up, and 63 patients were included in the current analysis (Figure 1).

There were 50 males and 13 females in this study, with a median age of 57.3 years (range, 39–70 years) at the first surgery and 60.3 years (range, 43–73 years) at the recurrence. The baseline features of the enrolled patients were listed in Table 1. Adenocarcinoma accounted for 46% of the cases (29/63), followed by squamous cell carcinoma (27/63, 42.9%). There were 45 (71.4%) patients diagnosed with pathologic stage I, 11 (17.5%) with stage II, and 7 (11.1%) with stage III by previous curative intent surgery. Among them, 53 patients (84.1%) were pathologically staged N0, 5 (7.9%) staged N1, and 5 (7.9%) staged N2. The gene mutation status was available for 17 patients, of whom 14 cases suffered from epidermal growth factor receptor (EGFR) mutation (22.2%), 1 case exhibited anaplastic lymphoma kinase (ALK) mutation (1.6%), and 2 cases presented Kirsten rat sarcoma viral oncogene (KRAS) mutation (3.2%). No patients received neoadjuvant therapy before the first-time surgery. All patients received PET-CT and brain MRI examinations for preoperative evaluation. The majority of the patients (81.0%) in stage IB–III, as well as 1 in stage IA because of sarcomatoid carcinoma, received postoperative adjuvant therapy, including 52 cases of chemotherapy and 1 case of chemoradiotherapy. The median interval between surgery and ipsilateral recurrence was 37.0 months (range 2–118 months). All patients underwent an anatomical surgical resection consisting of lobectomy (*n* = 48; 76.2%), sublobectomy (*n* = 6; 9.5%), or sleeve resection (*n* = 9; 14.3%). Surgical data for lobectomy and sublobectomy patients were summarized in Table 1.

**Table 1:** Patients’ characteristics (*n* = 63).

Characteristic	Number of patients (%)
Sex	
Male	50 (80.0)
Female	13 (20.0)
Age at first surgery, years	
Median (range)	57.3 (39–70)
Age at recurrence, years	
Median (range)	60.9 (43–73)
Operation center	
Shanghai Pulmonary Hospital	54 (85.7)
Other hospital	9 (14.3)
Characteristics of primary tumor	
Tumor location	
Left upper lobe	20 (31.7)
Left lower lobe	19 (30.2)
Right upper lobe	9 (14.3)
Right middle lobe	5 (8.0)
Right lower lobe	10 (15.9)
Type of first resection	
Sublobectomy	6 (9.5)
Lobectomy	48 (76.2)
Sleeve lobectomy	9 (14.3)
NSCLC histologic types	
Adenocarcinoma	29 (46.0)
Squamous cell carcinoma	27 (42.9)
Others	7 (11.1)
Genetic mutation	
Mutation	17 (27.0)
EGFR	14 (22.2)
ALK	1 (1.6)
KRAS	2 (3.2)
Wild-type	39 (61.9)
Unknown	7 (11.1)
Primary stage	
I	45 (71.4)
II	11 (17.5)
III	7 (11.1)
Adjuvant therapy	
Yes	52 (82.5)
No	11 (17.5)

NSCLC, non-small cell lung cancer; EGFR, epidermal growth factor receptor; ALK, anaplastic lymphoma kinase; KRAS, Kirsten rat sarcoma viral oncogene.

### Surgical procedure

**Figure 2:**
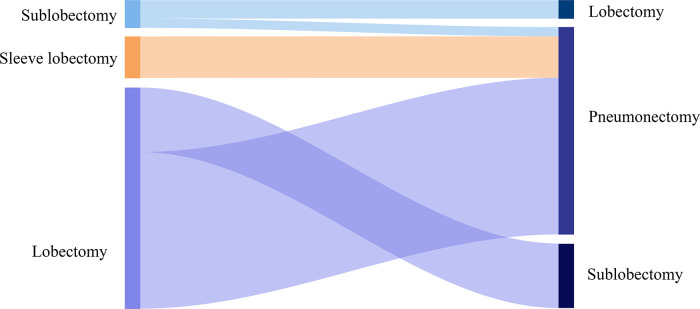
Sankey diagram of primary and completion surgery methods.

Of the enrolled cases, 24 (38.1%) patients suffered a right-sided and 39 (61.9%) patients a left-sided R0 completion residual lung resection (Table 2, Figure 2). Among the cases who received pneumonectomy, intrapericardial resection was performed to transect the pulmonary vein structures because of severe hilar fibrosis in three patients, chest wall and rib resection were adopted to treat the invasive lesion in two patients, and trachea carinaplasty was selected to treat the lesion near the main bronchus. The bronchial stump was covered with a muscle flap in seven cases. In the lobectomy group, no patients received intrapericardial resection while an additional resection was performed in six patients, including two cases of chest wall resection and one case of sleeve resection. The median number of dissected lymph nodes was three (range 1–5). The median length of ICU stay and in-hospital stays were 1.7 (range 0–10 days) and 8 days (range 3–18 days), respectively. In-hospital complications were observed in seven patients, including two cases of pulmonary embolism, two cases of pulmonary infection, one case of arrhythmia, one case of hemothorax, and one case of prolonged air leak. No patient died within 30 or 90 days after completion of residual lung resection, whereas 26 patients died because of disease progression.

**Table 2:** Characteristics of recurrence (*n* = 63).

Characteristic	Number of patients (%)
Interval (month) between surgery and recurrence	
Median (range)	41.5 (2–118)
Recurrence tumor location	
Left side	39 (61.9)
Right side	24 (38.1)
Type of second resection	
Pneumonectomy	45 (71.4)
Nonpneumonectomy	18 (28.6)
Sublobectomy	14 (22.2)
Lobectomy	4 (6.3)
Operation center	
Shanghai Pulmonary Hospital	63 (100.0)
Other hospital	0 (0.0)
Surgical approach	
VATS	12 (19.0)
Thoracotomy	46 (73.0)
Conversion to thoracotomy	5 (7.9)
Gross tumor size (mm)	
Median (range)	26 (7–75)
Number of retrieved lymph node stations (*n*)	
Median (range)	3.6 (1–7)
*p*-N classification at recurrence	
N0	53 (84.1)
N1	4 (6.3)
N2	6 (9.5)
r-Stage	
IIIA	57 (90.5)
IIIB	6 (9.5)
Treatment before or after second resection	
Adjuvant therapy	44 (69.8)
Neoadjuvant therapy	3 (4.8)
Hospital stay (day)	
Median (range)	8.2 (3–18)
In-hospital complication	
None	56 (88.9)
Pulmonary embolism	2 (3.2)
Pneumonia	2 (3.2)
Arrhythmia	1 (1.6)
Hemothorax	1 (1.6)
Air leakage for long time	1 (1.6)

VATS, video-assisted thoracoscopic surgery.

### Model specifications and predictors of PRS and OS

**Figure 3:**
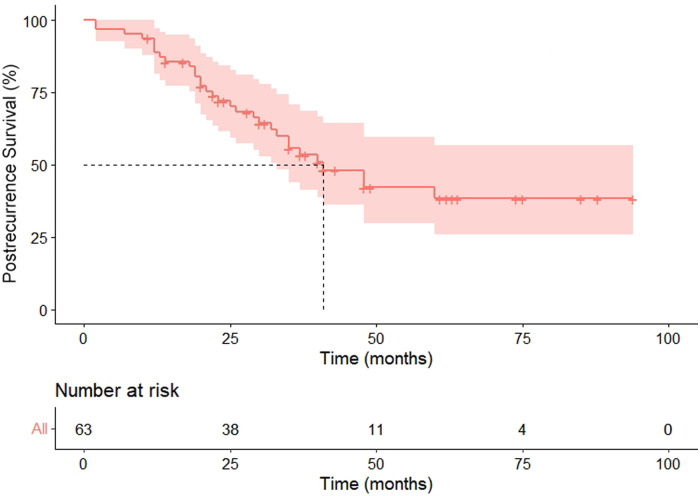
Postrecurrence survival of enrolled patients.

**Figure 4:**
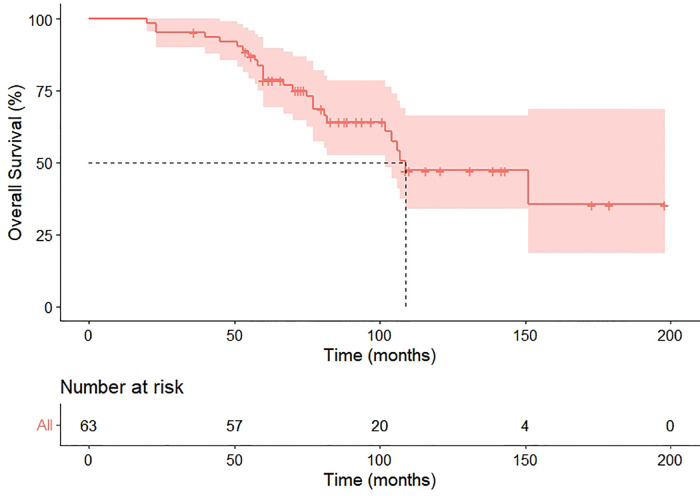
Overall survival of enrolled patients.

Within the median follow-up of 97 months (95% CI 80.4–113.6), a second recurrence was observed in 31 (49.2%) patients, including 25 metastasis and six locoregional recurrences, and 26 patients died because of the disease during follow-up. Progression in the pneumonectomy group was 20 (44.4%), all of which were distant metastasis. Our cohort exhibited the median PRS and OS were 41 months (HR 6.8, 95%CI 27.6–54.4) and 109 months (HR 17.3, 95%CI 75.1–142.9), respectively (Figures 3, 4). The 2-year and 5-year PRS rate was 55% and 36%, and the 2-year and the 5-year OS rate was 95% and 75%, respectively.

The demographic and clinical characteristics, together with tumor characteristics of clinical importance, were selected as candidate variables in prediction model 1. Then, backward stepwise selection using the AIC in the Cox proportional hazards regression model was used to identify the hazard factors of both PRS and OS in model 2. For PRS, model 2 included 3 variables: sex, r-stage, and adjuvant therapy after the second resection. Among them, the r-stage had the strongest association with recurrence risk, and patients with r-stage IIIB (HR, 3.35; 95% CI, 1.11–10.10; *P* = 0.03) faced higher risks in recurrence (Table 3). Similarly, two variables were included in model 2 for OS: age at recurrence and stage of the primary tumor, and the latter was the most significant hazard factor for OS. Patients with stage III after the first resection (HR, 6.26; 95% CI, 2.00–19.55; *P* < 0.01) indicated worse survival (Table 4).

**Table 3:** Cox proportional hazards regression model showing the association of variables with PRS.

	Model 1^a^		Model 2^b^	
	HR (95% CI)	*P*	HR (95% CI)	*P*
Sex		0.10		0.07
Male	1 (reference)		1 (reference)	
Female	0.30 (0.07–1.27)		0.37 (0.12–1.10)	
Age at recurrence (year)		0.61		
≤59	1 (reference)			
<59	0.80 (0.34–1.89)			
Comorbidity		0.87		
No	Reference			
Yes	1.09 (0.39–3.06)			
Histologic types				
Adenocarcinoma	1 (reference)			
Squamous cell carcinoma	2.03 (0.55–7.46)	0.29		
Others	0.99 (0.23–4.25)	0.98		
Genetic mutation				
Wild-type	1 (reference)			
Mutation	1.28 (0.33–4.97)	0.72		
Unknown	2.53 (0.55–11.74)	0.24		
Stage of primary tumor				
I	1 (reference)			
II	0.69 (0.23–2.08)	0.51		
III	2.88 (0.88–9.45)	0.08		
Type of second resection		0.09		
Nonpneumonectomy	1 (reference)			
Pneumonectomy	0.41 (0.15–1.13)			
r-Stage		0.14		0.03
IIIA	1 (reference)		1 (reference)	
IIIB	2.75 (0.72–10.57)		3.35 (1.11–10.10)	
Adjuvant therapy after second resection		0.40		0.11
No	1 (reference)		1 (reference)	
Yes	1.57 (0.55–4.49)		2.21 (0.84–5.80)	

r-stage, recurrence stage.

^a^ Model 1 included all the variables, and the AIC was 231.4.

^b^ Model 2 was filtered by Cox proportional hazards regression model, and the AIC was 211.2.

**Table 4:** Cox proportional hazards regression model showing the association of variables with OS figure legends.

	Model 1^a^		Model 2^b^	
	HR (95% CI)	*P*	HR (95% CI)	*P*
Sex		0.23		
Male	1 (reference)			
Female	0.38 (0.08–1.87)			
Age at recurrence (year)		0.23		0.08
≤59	1 (reference)		1 (reference)	
<59	0.55 (0.21–1.45)		0.46 (0.19–1.11)	
Comorbidity		0.76		
No	1 (reference)			
Yes	0.81 (0.21–3.07)			
Histologic types				
Adenocarcinoma	1 (reference)			
Squamous cell carcinoma	2.94 (0.54–16.10)	0.21		
Others	2.68 (0.63–11.43)	0.18		
Genetic mutation				
Wild-type	1 (reference)			
Mutation	1.17 (0.21–6.75)	0.85		
Unknown	2.30 (0.38–13.75)	0.36		
Stage of primary tumor				
I	1 (reference)		1 (reference)	
II	1.93 (0.57–6.57)	0.29	2.06 (0.72–5.85)	0.18
III	6.14 (1.67–22.62)	<0.01	6.26 (2.00–19.55)	<0.01
Type of second resection		0.22		
Nonpneumonectomy	1 (reference)			
Pneumonectomy	0.48 (0.15–1.55)			
r-Stage		0.30		
IIIA	1 (reference)			
IIIB	2.13 (0.52–8.71)			
Adjuvant therapy after second resection		0.57		
No	1 (reference)			
Yes	1.40 (0.44–4.50)			

r-stage, recurrence stage.

^a^ Model 1 included all the variables, and the AIC was 231.4.

^b^ Model 2 was filtered by Cox proportional hazards regression model, and the AIC was 211.2.

## Discussion

This retrospective study revealed that the postoperative complications and prognosis of completion pulmonary resection for lung cancer were acceptable. In patients affected by after-surgery NSCLC recurrence, survival is poor. Recurrence predisposing factors were: N1 lymph nodes metastasis, lymphatic vessels infiltration, and visceral pleura infiltration (10). The guidelines of the National Comprehensive Cancer Network published in 2014 recommended treating patients with surgery whenever possible if local recurrence is confirmed (11). On the contrary, unradical resectable cases should receive radiochemotherapy (12). We have observed that the 2-year PRS rate after complete resection was 55%; however, the 2-year OS rate was 95%. Moreover, we obtained a remarkable 5-year survival rate of 75% after the complete resection. These results suggest that the completion of residual lung resection could contribute to prolonged PRS and OS time through local disease control.

There is still a lack of investigation regarding the role of postoperative chemoradiotherapy on the completion of pulmonary resection. In this study, 84% of patients received chemotherapy or chemoradiotherapy prior to the complete pulmonary resection. For lung cancer patients experiencing progression after surgery, there are various ways of next-line systemic treatments including targeted therapy, cytotoxic chemotherapy, or immune checkpoint inhibitors (ICI) (13). However, if the number of recurrent tumors is limited, surgical resection, high-dose re-irradiation, and microwave ablation, or radio-frequency ablation may be selected (14–19). Gomez et al. prospectively reported that despite the limited case number, completion resection improved patient survival compared with maintenance therapy or observation in patients with recurrent NSCLC (14, 20). There is no standardized treatment plan for patients with local recurrence or residual lung tumor after the first surgery, but completion of pulmonary resection should be considered (21, 22). Moreover, our results suggest that independent of the NSCLC histologic subtype, complete residual lung resection achieves good outcomes and may be considered as the first choice for ipsilateral recurrence, and postoperative chemotherapy fails to significantly improve OS. Within the constraints of small sample size, this may be affected by various factors and further larger-scale investigation is needed to clarify the role of chemotherapy after complete pulmonary resection.

We showed that the stage of primary tumor was the only prognostic factor for OS after completion lung resection, and patients with stage III faced higher risks of recurrence. It was suggested that despite the r-TNM stage, stage III cancer may be prone to experience progression compared with patients in stage I. Poor response to chemotherapy may represent more resistant tumor clones, and the advanced stage of the primary tumor may also represent a higher tumor burden, so these factors might be prognostic and important to predict long-term survival even after complete surgical treatment (23, 24). Lobectomy is currently the standard surgery type for invasive lung cancer, and our results also showed that compared with sublobectomy, lobectomy in the first surgery exhibited a better prognosis even after complete resection. Survival after recurrence in NSCLC patients who received surgery is commonly unfavorable. PRS rates were first reported as 37% and 17% at 1 and 2 years, respectively, whereas they were calculated based on the data before 2001 (25, 26). Later, the PRS rates reached 68.3% at 1 year and 45.8% to 52.1% at 2 years when molecularly targeted therapies were introduced clinically (27). The improvements in PRS may be attributable to the introduction of molecular targeted therapies and immunotherapies, better selection of patients, and earlier detection of recurrence upon innovative diagnostic modalities. It was suggested occult metastasis may be existed during the first time of treatment, while sublobectomy may result in an underestimation of the stage. On the contrary, even though the r-TNM stage presented a good prediction for prognosis, different completion resection methods failed to show significance on either PRS or OS. The reason behind the prognostic potential of these factors suggests that the control of the primary tumor may be important to achieve cancer-free survival.

In terms of safety, the postoperative complication rate was 11.1% and the mortality rate was 0%, which suggested that completion surgery was not so risky. However, patients enrolled in this study were low-risk patients as most of the patients were relatively young (median age at completion resection = 60.3 years old) and there was no patient with interstitial lung disease. Considering this, the complication rate was still higher than in normal lobectomy or pneumonectomy cases and we must still pay attention to perioperative management. To prevent complications, we adopted several measures, such as prolonged perioperative prophylactic intravenous antibiotics to reduce the chance of infection; bronchial stump embedding to prevent bronchopleural fistula formation in high-risk patients, and aggressive postoperative mobilization or timely bronchoscopic suction to alleviate sputum stasis and atelectasis.

This study has several limitations. First of all has a retrospective nature, thus limiting the power of our evidence. We were unable to create a control group because the volume of patients that were followed up after primary resection for NSCLC in our hospital was too high. Of course, the comparison between surgically treated and nonsurgically treated patients with postoperative recurrent NSCLC would be ideal and could reveal the impact of the different treatment approaches on the oncological outcome. Moreover, for the same above-mentioned reason, it was difficult to obtain the recurrence rate of a patient surgically treated in our Institution during the same period, which may contribute to understanding the oncological outcome. In addition, the sample is small (*n* = 93) and heterogeneous in characteristics. Even if not included in reported data, patients that received completion pneumonectomy exhibited higher cardiopulmonary function and fitness than the nonpneumonectomy patients. This could have led to a selection bias. Finally, although patients with positive driver gene mutations were included, no one received molecular targeted therapies or immunotherapies, thus their prognostic role should be carefully interpreted.

## Conclusion

Completion pulmonary resection is an acceptable option in selected patients with recurrent lung cancer after surgery. The patients with r-stage earlier than IIIA may benefit from completion pulmonary resection but not IIIB. Completion pneumonectomy failed to significantly prolong the OS. The OS in the enrolled cases was mainly affected by the p-TNM stage assessed by the first resection for primary lung cancer.

## Data Availability

The raw data supporting the conclusions of this article will be made available by the authors, without undue reservation.
